# Arrhythmogenic Cardiomyopathy: from Preclinical Models to Genotype–phenotype Correlation and Pathophysiology

**DOI:** 10.1007/s12015-023-10615-0

**Published:** 2023-09-20

**Authors:** Xuehui Fan, Guoqiang Yang, Firat Duru, Maurizio Grilli, Ibrahim Akin, Xiaobo Zhou, Ardan Muammer Saguner, Ibrahim EI-Battrawy

**Affiliations:** 1 Key Laboratory of Medical Electrophysiology, Ministry of Education and Medical Electrophysiological Key Laboratory of Sichuan Province, Collaborative Innovation Center for Prevention of Cardiovascular Diseases, Institute of Cardiovascular Research, Southwest Medical University, Luzhou, Sichuan China; 2 Cardiology, Angiology, Haemostaseology, and Medical Intensive Care, Medical Centre Mannheim, Medical Faculty Mannheim, Heidelberg University, Heidelberg, Germany; 3 European Center for AngioScience (ECAS), German Center for Cardiovascular Research (DZHK) Partner Site Heidelberg/ Mannheim, and Centre for Cardiovascular Acute Medicine Mannheim (ZKAM), Medical Centre Mannheim, Heidelberg University, Partner Site, Heidelberg-Mannheim, Germany; 4 Department of Acupuncture and Rehabilitation, the Affiliated Traditional Chinese Medicine Hospital of Southwest Medical University, Luzhou, China; 5 Research Unit of Molecular Imaging Probes, Department of Radiologic Technology, Faculty of Associated Medical Sciences, Chiang Mai University, Chiang Mai, Thailand; 6 Department of Cardiology, University Heart Centre, University Hospital Zurich, Zurich, Switzerland; 7 Faculty of Medicine, University Medical Centre Mannheim (UMM), University of Heidelberg, Mannheim, Germany; 8 First Department of Medicine, University Medical Centre Mannheim, Theodor-Kutzer-Ufer 1-3, 68167 Mannheim, Germany; 9 Department of Cardiology and Angiology, Ruhr University, Bochum, Germany; Institute of Physiology, Department of Cellular and Translational Physiology and Institut für Forschung und Lehre (IFL), Molecular and Experimental Cardiology, Ruhr- University Bochum, Bochum, Germany

**Keywords:** Arrhythmogenic cardiomyopathy, Desmosomes, Animal models, Human-induced pluripotent stem cell-derived cardiomyocytes, Pathophysiology

## Abstract

Arrhythmogenic cardiomyopathy (ACM) is a hereditary myocardial disease characterized by the replacement of the ventricular myocardium with fibrous fatty deposits. ACM is usually inherited in an autosomal dominant pattern with variable penetrance and expressivity, which is mainly related to ventricular tachyarrhythmia and sudden cardiac death (SCD). Importantly, significant progress has been made in determining the genetic background of ACM due to the development of new techniques for genetic analysis. The exact molecular pathomechanism of ACM, however, is not completely clear and the genotype–phenotype correlations have not been fully elucidated, which are useful to predict the prognosis and treatment of ACM patients. Different gene-targeted and transgenic animal models, human-induced pluripotent stem cell-derived cardiomyocyte (hiPSC-CM) models, and heterologous expression systems have been developed. Here, this review aims to summarize preclinical ACM models and platforms promoting our understanding of the pathogenesis of ACM and assess their value in elucidating the ACM genotype–phenotype relationship.

**Graphical Abstract:**

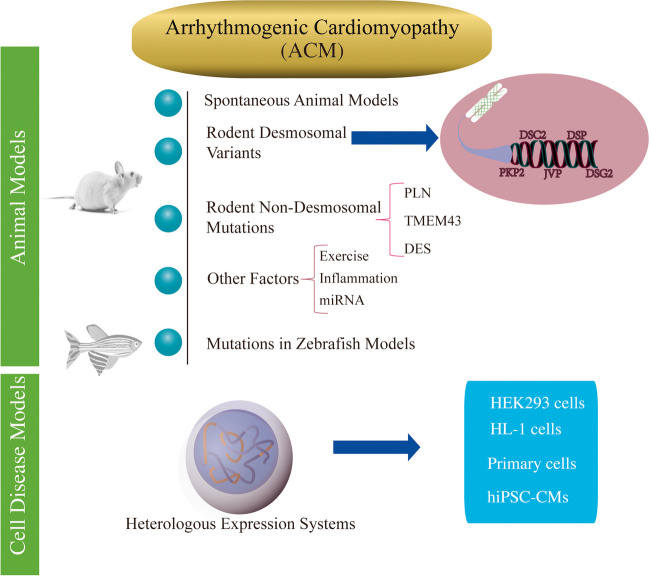

## Introduction

Arrhythmogenic cardiomyopathy (ACM), also known as arrhythmogenic right ventricular dysplasia (ARVD) or arrhythmogenic right ventricular cardiomyopathy (ARVC), is an inherited cardiac disease, which is characterized by fibro-fatty replacement of the myocardium, progressive ventricular dysfunction, thrombus formation and an increasingly high risk of sudden cardiac death (SCD) [1–4]. Despite right ventricular dysfunction being the predominant clinical manifestation, it has been recently proved that left ventricular abnormalities are observed in patients with ACM to some extent and may even be the initial manifestation of the disorder [5–10]. Fontaine et al. described ACM in detail for the first time in 1982 [11]. The incidence rate between males and females is about 2:1, especially in young adults and in athletes, probably due to sex hormones, and other factors, such as competitive sports, meteorological factors, intracardiac thrombosis, atrial arrhythmias as well as brady-arrhythmias [2, 8, 12–22].

**Fig. 1:**
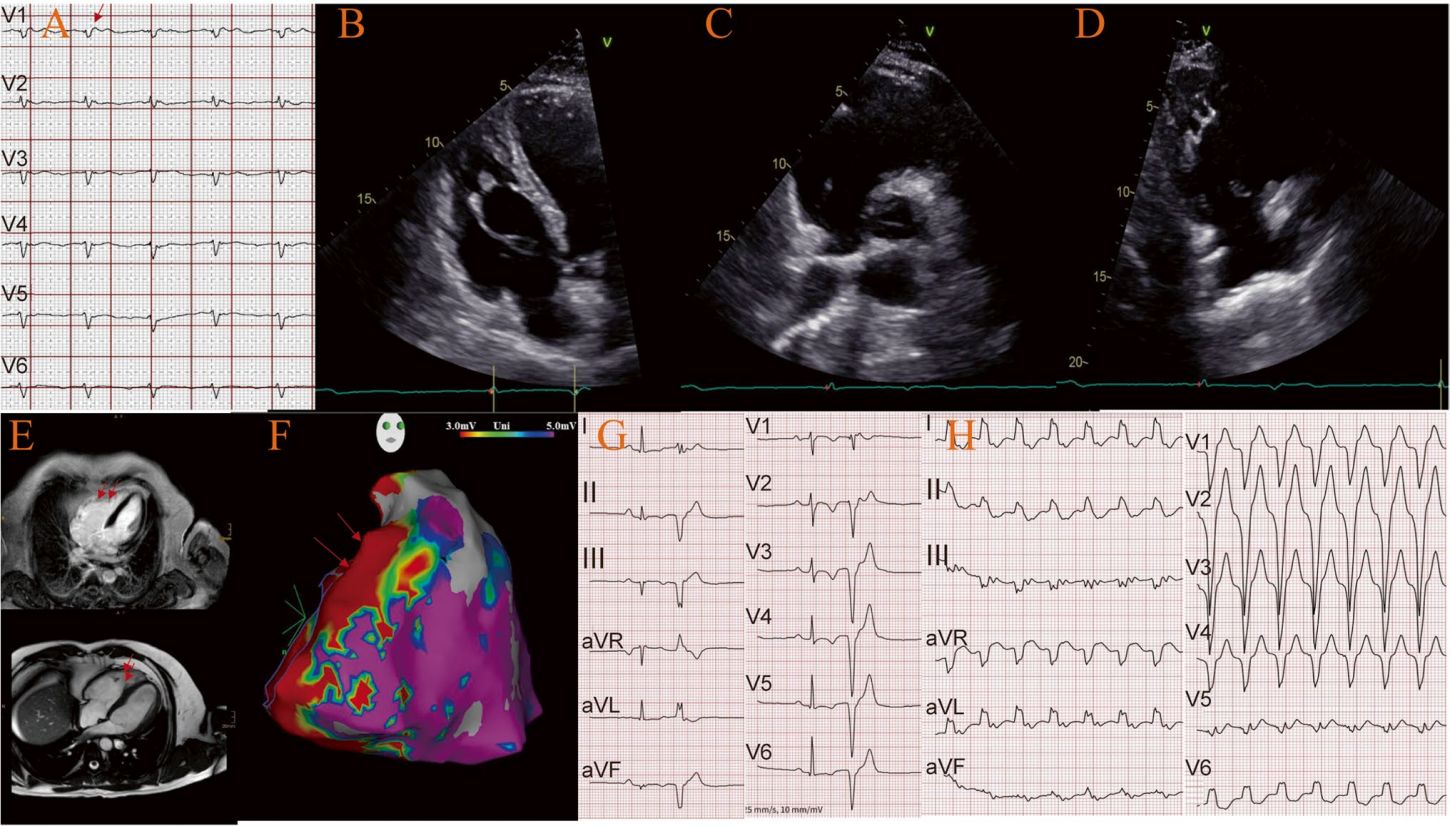
Illustrative cardiac MRI images, transthoracic echocardiography, 12-lead ECG with epsilon waves, left-bundle branch block VT with superior axis and 3-D endocardial RV mapping of patients with definite ACM harboring pathogenic desmosomal variants. A. 12-lead ECG of an ACM patient presenting with epsilon waves in right precordial leads harboring a pathogenic DSP variant; B-D. Transthoracic echocardiography in different views presenting RV/RVOT dilatation and regional wall thinning in a patient with ACM harboring a pathogenic PKP2 variant; E. Cardiac MRI presenting fibrosis, dilatation and regional wall thinning of the RV in the same patient; F. Endocardial 3D electro anatomical voltage mapping of the RV showing the typical “C-scar” in the subtricuspid region extending towards the RVOT in a patient with ACM harboring a pathogenic PKP-2 variant being referred for catheter ablation of VT; G.12-lead ECG presenting typical precordial T wave inversions and a ventricular premature beat from the RV in a patient with ACM harboring a pathogenic PKP-2 variant; H. 12-leadECG presenting a sustained ventricular tachycardia with LBBB morphology and superior axis originating in the subtricuspid area of the RV in a patient with ACM harboring a pathogenic PKP2 variant

**Fig. 2:**
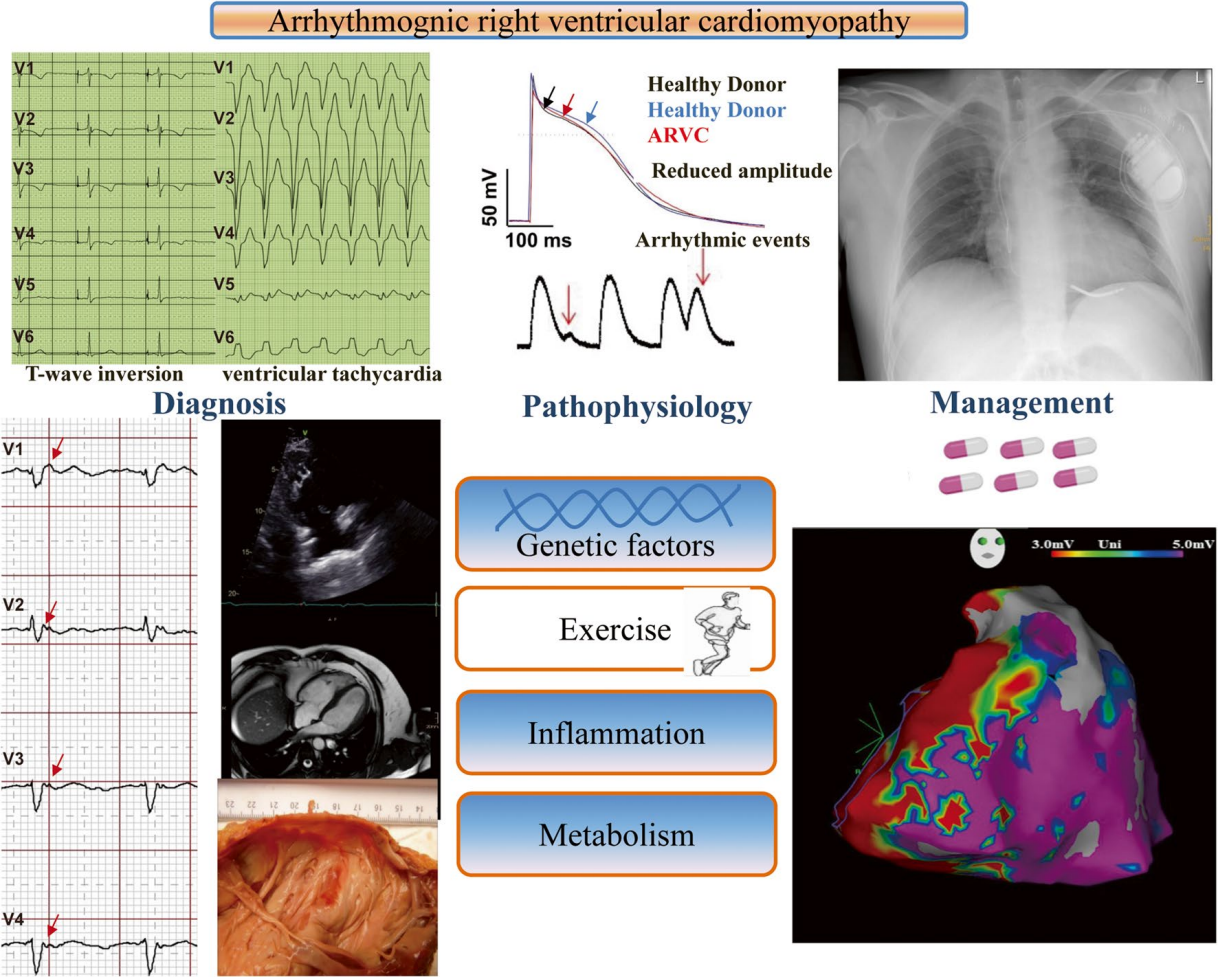
Diagnosis, pathogenesis, and management of ACM

The definite diagnosis of ACM is established based on major and minor clinical, electrocardiography, and echocardiography and cardiac magnetic resonance (CMR), genetic studies, biopsy, and histology [23, 24] (Fig. 1). Pathogenic variants in ACM-associated genes are the main diagnostic criteria. In 2021, Gasperetti et al. reported that reduced left ventricular ejection fraction (LVEF), advanced atrioventricular block (AVB), prolonged PR interval, longer QRS duration, right ventricular apical involvement, and positive [18] F-FDG PET scan exist in cardiac sarcoidosis (CS) mimicking ACM, whereas larger right ventricular outflow tract (RVOT) dimensions, subtricuspid involvement and T-wave inversions (TWIs) help to diagnose hereditary ACM [25]. In some cases, low specific electrocardiographic abnormalities, difficulties in interpretation imaging to assess right ventricular structure and function, multiple reasons for right ventricular arrhythmias, and the puzzling genetic testing make the diagnosis challenging [1] (Fig. 2). Importantly, there is no curative treatment for this life-threatening disease [26].

To date, more than 10 ACM-associated genes have been identified [27] (Table 1). Desmosomes in the heart contain five proteins, including junctional plakoglobin encoded by *JUP*, plakophilin-2 (PKP2) by *PKP2* which is the most common genetic cause of ACM, desmoplakin by *DSP*, desmoglein-2 by *DSG2*, and desmocollin-2 by *DSC2* [27–32]. There are also forms of autosomal recessive inheritance. Homozygous mutations in plakoglobin cause Naxos disease, which affects the heart and skin, and symptoms begin in childhood. Other forms of autosomal recessive inheritance are Carvajal syndrome caused by mutations in *DSP*, which is characterized by woolly hair, palmoplantar keratosis, and heart disease [1], and the homozygous p.Gln554X variant in *DSC2*, which is prevalent in around 10% of the Hutterite population and causes severe ACM.

**Table 1:** Genes associated with ACM

Gene	Chromosomal location	Encoded protein	Reference
DSP	6p24.3	Desmoplakin	Rampazzo et al. [28]
PKP2	12p11.21	Plakophilin-2	Gerull et al. [29]
DSG2	18q12.1	Desmoglein-2	Pilichou et al. [30]
DSC2	18q12.1	Desmocollin-2	Syrris et al. [31]
JUP	17q21.2	Junction plakoglobin	McKoy et al. [32]
DES	2q35	Desmin	Klaukeet al. [33]
TTN	2q31.2	Titin	Taylor et al. [34]
FLNC	7q32.1	Filamin C	Hall et al. [35]
LMNA	1q22	Lamin A/C	Quarta et al. [36]
TMEM43	3p25.1	Transmembrane protein 43	Merner et al. [37]
ACTN2	1q43	Actinin alpha 2	Good et al. [38]
LDB3	10q23.2	LIM domain binding 3	Lopez-Ayala et al. [39]
PLN	6q22.31	Phospholamban	Zwaag et al. [40]
SCN5A	3p22.2	Sodium voltage-gated channel alpha subunit 5	Erkapic et al. [41]
TGFβ3	14q24.3	Transforming growth factor beta 3	Beffagnaet al. [42]
CDH2	18q12.1	Cadherin 2	Mayosi et al. [43]
TJP1	15q13.1	Tight junction protein 1	Bortoli et al. [44]
CTNNA3	10q21.3	Catenin alpha 3	Hengel et al. [45]

In addition to mutations in desmosomal genes, an increasing number of non-desmosomal genes, such as desmin (*DES*), titin (*TTN*), lamin A/C (*LMNA*), Phospholamban (*PLN*), Transmembrane Protein 43 (*TMEM43*), and the sodium channel Nav1.5 (*SCN5A*) have also been reported as causative genes for ACM by using candidate gene sequence methods and linkage analysis, as inherited mutations identified in humans with cardiomyopathy are now modeled in transgenic or knock-in animals [46, 47]. In addition, at least 30–50% of ACM cases are also facilitated by non-genetic factors such as inflammation or exercise (Fig. 2) [48–51]. However, James et al. reported that only 8 genes including *PKP2, DSP, DSG2, DSC2, JUP, TMEM43, PLN,* and *DES*, had definitive or moderate evidence for ACM using the Clinical Genome Resource approach to gene-disease curation [52]. Therefore, understanding its genetic architecture and molecular mechanism, which will need to be fully elucidated by larger studies, may be helpful to improve the treatment of arrhythmias and prevent sudden cardiac death in ACM.

Unfortunately, the exact pathophysiological mechanisms and treatment strategies of ACM are still unclear. Preclinical models such as animal models, heterologous expression systems, and human cardiomyocytes derived from induced pluripotent stem cells (hiPSC-CMs) have been used to model the disease and pave the way to clarify the genotype–phenotype associations and the underlying mechanisms. This review summarizes the role of preclinical models in elucidating the pathophysiological mechanism of ACM, and the association between genotype and phenotype to provide evidence-based personalized management.

## Experimental Models of ACM

Presently, roughly half of ACM patients with the clinical phenotype have at least one identifiable genetic variant. This raises the possibility that genetic data could be used to predict disease severity [53]. Over the last 10 years, to gain a clear and in-depth understanding of the pathological mechanisms of ACM, researchers established diverse models in vivo and vitro to investigate the pathogenesis and clinical features of ACM development.

### Animal Models

To date, transgenic overexpression of genes and targeted genetic deletion animal models have been established to provide compelling evidence for elucidating the mechanisms of ACM development, which contributed to a better understanding of the pathophysiological processes. Furthermore, due to the developing genetic animal models harboring a clear genetic background, they are very suitable for serving as important tools to help uncover mechanisms that commonly and specifically underlie these disease phenotypes under controlled and standardized conditions.

#### Spontaneous Animal Models

For many years, people have noticed that boxer dog breeds are susceptible to right ventricular structural abnormalities, fibrofatty replacement, myocarditis, apoptosis, ventricular arrhythmia, and SCD, which means this model constitutes a new and potentially useful research tool that can be used to understand the complex clinical and pathogenic mechanisms of ACM disease, but the underlying diseases that lead to these clinical features have not been fully determined, so a series of related studies have been conducted on dog models. Furthermore, Boxers with ACM had a significant increase in serum cTnI concentration, suggesting that cTnI may be an indicator of stage or severity of ACM [54]. Also, Philip and coworkers identified clinically relevant cardiomyopathy in the common domestic cat having clinical features like right-sided congestive heart failure, supraventricular tachyarrhythmias, ventricular tachycardia, polymorphic ventricular arrhythmias, and right bundle-branch block, closely resembling human ACM [55–57]. The clinical features of ACM in dog and cat models can be observed, but they are all spontaneous models, which have certain limitations in studying the pathophysiological mechanism of ACM. Transgenic animals can be used to study the role of specific genes associated with ACM in the pathophysiology of ACM. Therefore, the construction of a transgenic mouse model that mimics the pathological characteristics of ACM through transplantation or gene-editing technology helped to further clarify the research on the pathogenesis of ACM.

#### Desmosomal Variants in Rodent Models

The cardiac desmosomal cell–cell junction protein complex is made up of five classic proteins. Genetic mutations in the desmosome genes that cause the destabilization/breakdown of the desmosome proteome are the core hallmarks of all genetic-based desmosome-targeted diseases including ACM [58–61]. Desmosomes are indirectly related to the electrochemical coupling of cardiomyocytes and signal transduction [62, 63]. Currently, the electrophysiological and pathological mechanisms caused by desmosomal protein defects are complex. In addition, electrophysiological abnormalities occur prior to major structural changes such as fibrosis in both the human disease and a murine model as a result of mislocalization and a reduction in expression of connexin-43 (Cx-43), leading to a slowed conduction [64, 65]. Different transgenic, knock-in, and conditional cardiac-specific mice models for the desmosomal genes have been developed to investigate the specific pathophysiological mechanism of ACM (Table 2).

**Table 2:** Gene, mutation, key findings, references in animal models studying ACM

	Animal	Gene	Mutation	Protein	Key findings
Spontaneous models	Boxer dogs [66–69]	-	-	-	Boxer dog breeds are susceptible to right ventricular (RV) structure abnormal, fibrofatty replacement, myocarditis as well as apoptosis, ventricular arrhythmia and SCD
	Cat [55–57]	-	-	-	The domestic cat has the following clinical features, like right-sided congestive heart failure, supraventricular tachyarrhythmias, ventricular tachycardia, polymorphic ventricular arrhythmias, and right bundle-branch block, closely resembling human ACM
Desmosomal mutationin transgenic rodents models	PKP2- heterozygous- null (PKP2-Hz) mice [70]	PKP2		plakophilin-2	Decreased amplitude and a shift in gating and kinetics of I_Na_
	PKP2 R735X mutation mice [71]		R735X		Impaired global RV systolic function and RV regional wall motion abnormalities
	PKP2-truncated mice [72]		Express the first 329 amino acids		Ultrastructural defects without fibro-fatty replacement, a dose-dependent ventricular arrhythmia susceptibility and CX43 and Na_v_1.5 remodeling and reduction
	PKP2cKO mice [70, 73–76]				(1) Lack of PKP2 can cause arrhythmia in a structurally normal heart (2) Istradefylline (an adenosine 2A receptor blocker) mitigated the progression of fibrosis and mechanical failure observed in PKP2cKO mice (3) The 2B adenosine receptor blocker PSB115 showed the opposite effect (4) Loss of PKP2 disrupted RV-predominant Ca^2+^ homeostasis prior to cardiomyopathy (5) There is a relationship between the abundance of PKP2 transcripts and the expression of transcripts that encode inflammation and immune response-related proteins in cardiomyocytes
	PKP2^+/-^ Mice [77]				Low-intensity workout does not cause an increase of sudden cardiac death and overt arrhythmia in a mouse model and seems to be even beneficial during an early, transient phase
	Heterozygous PKP2 knock-out mice (PKP2-Hz) [78]				(1) PKP2 haploinsufficient mice displayed reduced Ca2 + -handling-related proteins expression, such as CaV1.2, SERCA2a, AnkB, and Casq2 (2) Pressure overload increased levels of fibrosis and impaired electrical conduction rather than structural remodeling leading to exercise-induced pro-arrhythmic cardiac remodeling
	Heterozygousplakoglobin (PG) -deficientmice(plako( ±)) [79–82]	JUP		plakoglobin	(1) Increased right ventricular volume, reduced right ventricular function, and spontaneous ventricular ectopy, Unaltered left ventricular size and function (2) Therapy prevents RV enlargement and increased Cx43 expression in trained plako + /– mice
	cardiomyocyte-restricted *Jup* knockout mice [83]				Left and right ventricular dilation and aneurysm, cardiac fibrosis, cardiac dysfunction and spontaneous ventricular arrhythmias, increased β-catenin at adherens junctions
	Plakoglobin CKO mice [84]				Progressive loss of cardiac myocytes, extensive inflammatory infiltration, fibrous tissue replacement, and cardiac dysfunction, increased β-catenin stabilization; pathological hypertrophy
	cardiac- restricted expression of N271S-*dsg2* in mice [85, 86]	DSG2	N271S	desmoglein 2	Thinning of the left and right ventricular free walls, dilation of the right and left ventricles and biventricular aneurysms, Significant reduction in RV, LV and interventricular septal thickness, myocyte necrosis, progressive myocardial damage; a in vivo interaction between Dsg2 and the Na ( +) channel protein Na(V)1.5
	DSG2- mutant mice [87]		deletion of exons 4–6		(1) Increased mRNA expression of c-myc, ANF, BNF, CTGF and GDF15, (2) dilation of the left ventricle, (3) Early slowed left ventricular filling, (4) Increased systolic left ventricular diameter and systolic left ventricular end volume
	DSG2 mutant mice [88]				Dying cardiomyocytes with calcification appeared in lesions of all ages and lesions of young mutant mice and the older animals harbored high amounts of CD45 + immune cells
	*Dsg2*^*mt/mt*^ and *Dsg2*^*cKO/cKO*^ mice [89]				*Acta1* overexpression
	DSG2 mutation mice [90]		Q558*		Signs of fibrosis, decrease in desmosomal size and number, and reduction of the Wnt/β-catenin signaling in transgenic hearts
	homozygous knock-in mutant desmoglein-2 (Dsg2mt/mt) mice [91]				Impaired left ventricular function and extracellular vesicle treatment improved cardiac function, reduced cardiac inflammation, and suppressed arrhythmogenesis in ACM
	cardiac-restricted DP-deficient mice [92]	DSP		desmoplakin	Excess adipocytes and fibrosis in the myocardium, increased myocyte apoptosis, cardiac dysfunction, and ventricular arrhythmias
	DSP ( ±) mice [64]				Affecting myocyte electrical coupling, primarily Cx43 mislocalization, and promoting structural changes
	Cardiac-restricted DSP transgenic (Tg) mice [93]		V30M or Q90R, R2834H, W233X		Cardiomyocyte apoptosis, cardiac fibrosis, and cardiac dysfunction during adulthood
	cardiomyocyte-specific desmoplakin-deficient mice (DSP-cKO) [94]				Early ultrastructural defects in desmosomal integrity; biventricular dysfunction, failure and premature death; ventricular arrhythmias
	DSP (R2834H) mice [95]		R2834H		Endurance exercise accelerates AVC pathogenesis in Tg-DSP(R2834H) mice, Mutant mice have enlarged RV and reduced LV volume after exercise
	the G790del KI mice [96]	DSC2	G790del	desmocollin-2	Slight contractile dysfunction and Ca^2+^ dysregulation in the LV
	transgenic mice overexpressing DSC2 [97]	-	-	-	Cardiac specific overexpression of desmocollin-2 induces necrosis, acute inflammation and patchy cardiac fibrotic remodeling causing fulminant biventricular cardiomyopathy
Non-desmosomal mutation in transgenic rodents models	Overexpressing the mutant PLN-R14Del mice [98]	PLN	R14Del	phospholamban	cardiac hypertrophy, myocardial fibrosis, and premature death
	PLN-R14^Δ/Δ^ Mice and PLN-R14^Δ/^ Mice [99]		R420W		PLN-R14^Δ/Δ^ mice accelerated manner in less than 2 months, whereasPLN-R14^Δ/+^ mice exhibit cardiomyopathy at middle age
	the heterozygous hPLN^R14del^ (R14del) mutation [100]	PLN DES	R14Del	phospholamban desmin	Primary electric remodeling and increased arrhythmia susceptibility, interventricular repolarization gradients
	desmin knockout (*Des*^*−/−*^) mice [101]				Lepirudin reduced myocardial injury;
	β1D knockout (β1D^−/−^) mouse model [102]				(1) Integrin β1D deficiency and RyR2 Ser-2030 hyperphosphorylation in left ventricular tissue (2) Integrin β1D protein is significantly downregulated in ACM patient heart tissue in parallel with reduction of desmosomal proteins such as DSP, DSC2 and DSG2 (3) Exogenous integrin β1D decreases PKA-induced RyR2 Ser-2030 phosphorylation and RyR2 channel open probability
	TMEM43 S358L knock-in mice [103–105]	TMEM43	S358L		(1) Structural abnormalities and cardiac fibrofatty; (2) TMEM43mut mice treated with enalapril showed a significantly increased survival rate, and showed increased left ventricular ejection fraction, shortened QRS duration, and decreased left ventricular fibrosis at 4 months of enalapril treatment; (3) myocardial fibrofatty replacement and die at a very young age
	mice with Rho-kinase inhibition [106]	-	-	-	(1) Increased RV and LV dimensions and reduced LV ejection fraction and fractional shortening in DN-RhoK mice (2) Cardiac dilatation and dysfunction, myocardial fibrofatty changes, and ventricular arrhythmias
Other factors in rodent models	KK/Rvd mice [107]	-	-	-	Early cell death, alteration of the chromatin architecture
	*Sorbs2* knock-out mice [108]	-	-	-	Phenotypes of right ventricular dilation, right ventricular dysfunction, spontaneous ventricular tachycardia, and premature deaths; decreased cardiac function in both RV and LV
	Myh6-Cre: DspW/F mice [109]	-	-	-	Restored transcript levels of the majority of dysregulated genes in cardiac myocytes, reduced myocardial apoptosis, and induced eccentric cardiac hypertrophy
	*Csn6*-KO mice [110]	-	-	-	Cardiac *Csn6*-KO mice could recapitulate all classic disease features of ARVD/C
Zebrafish models	Knockdown of *dsc2* in zebrafish [111]	DSC2	-	desmocollin-2	Myocardial desmosome structure and contractility
	PKP2 knock-down zebrafish [112]	PKP2	-	plakophilin-2	Cardiac oedema, blood pooling, failure of the heart to loop, decreased heart rate and abnormal desmosomes
	Dsp-deficient zebrafish models [113]	DSP	-	desmoplakin	Desmosomes structure, Wnt/β-catenin, TGFβ/Smad3, and Hippo/YAP-TAZ signaling pathways affected
	zebrafish model [114]	DSC2	-	desmocollin-2	The R132C substitution impairs DSC2 function

##### PKP2

One of the most commonly disrupted genes in ACM is *PKP2* [1, 115]. Worth noting, even in a structurally normal heart, variants in human *PKP2* can cause life-threatening arrhythmias. Surprisingly, Moncayo-Arlandi et al. observed structural but not electrophysiological abnormalities in trained and old *PKP2*-truncated mice [72]. Of note, the reduced *PKP2* expression reduced Na current peak density and also altered channel kinetics as well as significantly reduced conduction velocity (CV) in cultured rat neonatal cardiomyocytes, suggesting that desmosomal proteins also directly interact with ion channels at the intercalated disc [70, 115, 116]. However, the signaling between loss of *PKP2* expression and structural cardiomyopathy is still unclear. Cerrone et al. thus investigated the role of ATP/adenosine in *PKP2* cKO mice models, finding that an adenosine 2A receptor blocker-Istradefylline mitigated the progression of fibrosis and mechanical failure, while PSB115 (a blocker of the 2B adenosine receptor) had opposite effects [73], indicative of involvement of adenosine receptor signaling. Kim and coworkers, using *PKP2* cKO mice 14 days post-tamoxifen (post-TAM) to explore early molecular/cellular events, found that loss of *PKP2* disrupted right ventricular-predominant Ca2 + homeostasis prior to cardiomyopathy, which is at least partly mediated by a Cx43-dependent permeability and inhibited by protein kinase C (PKC) inhibitors [74]. In conclusion, the loss of PKP2 led to electrophysiological abnormalities and ultrastructural defects.

##### JUP

To test if reduced desmosomal protein expression causes ACM, Kirchhof et al. for the first time found that heterozygous plakoglobin-deficient mice (plakoglobin^+/−^) showed an increased right ventricular volume, reduced right ventricular function, and spontaneous ventricular ectopy, which was accelerated by endurance training [79–81]. This suggests that plakoglobin deficiency provokes the functional phenotype of ACM. In addition, overexpression of the N-terminal mutants in embryonic mouse hearts can lead to embryonic lethality resembling that seen in *JUP*- and *PKP2*-deficient mice [79, 93, 117]. Fabritz et al. confirmed that load-reducing therapy prevents training-induced development of ACM in plako ( ±) mice [82]. Plakoglobin functions as a signaling protein via its ability to modulate the Wnt/β-catenin signaling pathway. However, Li et al. generated cardiac-restricted *Jup* knockout mice which largely recapitulated the clinical manifestation of human ACM, including ventricular dilation and aneurysm, cardiac fibrosis, cardiac dysfunction and spontaneous ventricular arrhythmias and increased β-catenin at adherens junctions, but there was no abnormality in Wnt/β-catenin signaling [83, 118]. Indeed, they also found cardiomyocyte death in *Jup* mutant hearts and the cell death was usually confined to the area close to the fibrosis replacement area [83]. Similarly, ablation of plakoglobin caused an increase in β-catenin stabilization associated with activated AKT and an inhibition of glycogen synthase kinase 3β, which may contribute to ACM pathogenesis [84]. They also demonstrated for the first time the dual functions of plakoglobin as a cell adhesion and signaling molecule in the working myocardium [84]. Similarly, the plakoglobin homolog, β-catenin was increased in a plakoglobin hypomorph (PG FN/Δ) hearts [119].

##### DSG2

In 2009, Pilichou et al. demonstrated for the first time that myocyte necrosis as the key initiator of myocardial injury triggered progressive myocardial damage, including an inflammatory response and massive calcification within the myocardium, followed by injury repair with fibrous tissue replacement, and myocardial atrophy [85]. They were also interested in whether mutation-induced intercalated disc remodeling impacts electrophysiological properties before the onset of cell death and fibrosis. Understanding the electrical remodeling in the early stage of ACM is crucial to elucidate mechanisms for ventricular arrhythmias. Therefore, they employed the same model, finding that a *DSG2* variant in a structural component of cardiac desmosomes affects ventricular conduction and arrhythmia susceptibility even prior to the onset of necrosis and replacement fibrosis due to a reduced I_Na_ density during the early ACM stages [86]. Inhibiting lectin, galactoside-binding, soluble, 3 (Lgals3) in zebrafish reduced Wnt and TGF-β signaling, increased Hippo/YAP-TAZ signaling, and induced alterations in desmosome integrity and stability [120].

Mutant mice lacking a part of the extracellular adhesive domain of *DSG2* developed ventricular dilation leading to cardiac insufficiency and eventually premature death, which are correlated with increased mRNA expression of c-myc, ANF, BNF, CTGF, and GDF15, markers for cardiac stress, remodeling and heart failure [87]. To unravel the sequence of myocardial alterations during ACM onset and progression, histological analyses were performed on the hearts of *DSG2* mutant mice from the juvenile to the adult state, suggesting that dying cardiomyocytes with calcification appeared in lesions of all ages and lesions of young mutant mice and the older animals harbored high amounts of CD45 + immune cells [88]. The expression of α1-skeletal muscle actin (*Acta1*) was upregulated in the myocardium of *Dsg2*^*mt/mt*^ and *Dsg2*^*cKO/cKO*^ mice. Its early upregulation is related to the impaired mechanical coupling of cardiomyocytes, and the later stage is the production of fibrotic cardiomyopathy and adjacent cardiomyocytes caused by *TGFβ* [89]. The stabilization of *Dsg2* binding by a linking peptide (Dsg2-LP), serving as a novel approach to treat arrhythmia in patients with AC, is efficient to rescue arrhythmia in an AC mouse model, disrupted cohesion induced by siRNA-mediated plakoglobin or *Dsg2* depletion as well as Cx-43 mislocalization and conduction irregularities [121].

##### DSP

Subunit 6 of the cardiac constitutive photomorphogenesis 9 (COP9) signalosome (CSN6), a component of the cardiac desmosome complex, directly interacts with the N-terminus of DSP [110]. Furthermore, hearts from *Dsp*-cKO mouse model displayed reduced junctional localization of CSN6 and similar protein degradation defects, which is consistent with loss of CSN6 function [110]. Using Cardiac-restricted *DSP* transgenic mice, Yang et al. identified 4 novel mutations in *DSP*, like a nonsense mutation in the N terminus of *DSP* (W233X) leading to haploinsufficiency, two missense mutations in the N terminus of *DSP* (V30M and Q90R) affecting the normal localization of *DSP* in vitro probably due to loss of binding to *JUP* as well as the overexpression of a C-terminal *DSP* mutation (R2834H) leading to cardiac defects [93]. In addition, studies have linked mutations in the desmosomal protein and desmoplakin to patients primarily exhibiting left ventricular-dominant and biventricular forms of ACM [19, 122, 123]. Homozygous *DSP*-cKO mice display early ultrastructural defects in desmosomal integrity leading to a cardiomyopathy reminiscent of a biventricular form of ACM, which includes cell death and fibro-fatty replacement within the ventricle leading to biventricular dysfunction, failure and premature death [94]. Exercise and catecholamine stimulation exacerbated ventricular arrhythmias that happened in *DSP*-cKO mice, whose hearts exhibited right ventricular conduction defects related to loss of connexin 40 expression and electrical wavefront propagation defects associated with loss of Cx43 expression [94].

##### DSC2

To date, there are few transgenic or mutant *DSC2* knock-in mouse models. It has been reported that the graded knockdown of *DSC2* in zebrafish embryos can severely disrupt myocardial desmosome structure and contractility. Also, a heterozygous mutation (c.631-2A → G) in *DSC2* is a cause of familial ACM11 [111]. However, the G790del mutation in a *DSC2* knock-in mouse model showed a slight contractile dysfunction and Ca^2+^ dysregulation in the left ventricular, which was not relevant to the pathogenesis of ACM, perhaps because G790del in *DSC2* alone is insufficient to develop ACM in mice [96]. Other non-genetic factors such as microRNAs may contribute to the down-regulation of desmosomal protein that causes desmosome dysfunction [124]. The overexpression of miR-130a in adult myocardium would promote downregulation of *DSC2* and lead to a disease phenotype resembling AC, suggesting that αMHC-tTA/TetO-miR130a mice may serve as a potential model to study ACM [125].

#### Non-desmosomal Mutations in Rodent Models

Currently, several rare mutations in non-desmosome genes have been found in human ACM patients, including cardiac electrophysiology (encoded by *SCN5A*, *PLN*), Z-band proteins (encoded by *DES*, *LDB3*, *ACTN2*), nuclear envelope proteins (encoded by *TMEM43*, *LMNA*, *LEMD2*), or proteins involved in cell–cell or cell to extracellular matrix (ECM) adhesion (encoded by *CTNNA3*, *CDH2, TJP1*, *ILK*, *FLNC*). However, there are currently a few specific mice or zebrafish models available for ACM-associated non-desmosomal mutations.

##### PLN

Phospholamban encoded by the *PLN* gene is present in the sarcoplasmic reticulum membrane, regulating calcium handling by reversibly inhibiting the activity of the sarcoplasmic reticulum calcium ATPase 2 (SERCA2) [126]. Animal models have been established to mimic human ACM. Overexpression of Arginine (Arg) 14 deletion (*PLN*-R14del) in mice resulted in super inhibition of SERCA, which may be related to cardiac hypertrophy, myocardial fibrosis, and premature death. PLN ablation (*PLN*-KO) significantly increases cardiac contractile parameters, whereas overexpressing *PLN* inhibits cardiac function [127, 128]. A mutation in the *PLN*-R14Del may lead to mis-localization of *PLN* from SR to the sarcolemma and increased Na/K-ATPase (NKA) activity [129]. *PLN*-R14^Δ/Δ^ mice accelerated manner in less than 2 months, whereas *PLN*-R14^Δ/+^ mice exhibit cardiomyopathy at middle age [99]. Raad et al. found that R14del hearts exhibited increased arrhythmia susceptibility at the early stages of the disease, which provide an electrophysiological basis for the typical mode of SCD in these patients [100].

##### TMEM43

Transmembrane protein 43 (TMEM43) localized mostly at the nuclear membrane is related to a highly lethal and fully penetrant ACM subtype, which is called ARVD5[MIM:604400] [37, 103, 130–133]. The Ser358Leu mutation of *TMEM43* knock-in mice displays ACM-like phenotypes, such as higher level of left ventricle end-diastolic dimension (LVEDD), lower level of posterior wall thickness in systole (PWTS) as well as cardiac fibrosis and adipogenesis [104]. Of note, *TMEM43* S358L mutation up-regulated nuclear factor-κB (NF-κB)-TGFβ signal cascade during ACM cardiac fibrosis, revealing the regulatory mechanism of ACM development [104]. Barthe et al. demonstrated that transgenic mice expressing *TMEM43*-S358L exhibited myocardial fibrofatty replacement and died at a very young age whereas GSK3β inhibitor or overexpression of calcineurin Aβ1 in *TMEM43* mutant mice can improve cardiac function but antifibrotic treatment can’t, suggesting that a new therapeutic approach could be used in ACM5 patients in the future [103]. *TMEM43*-mutant mice treated with enalapril showed a significantly increased survival rate, and showed increased left ventricular ejection fraction, shortened QRS duration, and decreased left ventricular fibrosis at 4 months of enalapril treatment whereas metoprolol did not show positive effects [105], suggesting that enalapril can preventively treat asymptomatic ACM5 gene carriers.

##### DES

The prevalence of *Des* mutations in ACM is higher than previously described, estimated at 2–3%, and *Des*^*−/−*^ mice recapitulate most of the pathognomonic features of ACM [46, 134]. The crosstalk between the complement and coagulation systems exacerbated the myocardial injury of ACM mice which was alleviated by using the thrombin inhibitor lepirudin [101]. *Des* elimination leads to structural and functional abnormalities of the sinoatrial pacemaker complex (SANcl) [101]. As such, clarifying the molecular correlation between coagulation and the complement system may provide potential and new molecular therapeutic targets for ACM to improve clinical outcomes.

#### Other Factors in Rodent Models

To date, due to low and uncertain ACM penetrance, it is crucial to understand the cardiac remodeling caused by environmental stressors.

##### Exercise

In addition, mice with Rho-kinase inhibition in the developing heart (SM22α-restricted) spontaneously presented cardiac dilatation and dysfunction, myocardial fibrofatty changes, and ventricular arrhythmias, which further led to premature sudden death, phenotypes consistent with the characteristics of ACM in humans, demonstrating a novel crucial role of Rho-kinase inhibition during cardiac development in the pathogenesis of ACM [106]. Physical exercise has been observed as the common denominator in provoking an arrhythmic phenotype. Physical exercise has been observed to cause arrhythmia phenotype [135]. However, treadmill exercise may restore the transcription levels of most dysregulated genes in cardiomyocytes, reduce cardiomyocyte apoptosis, and induce eccentric cardiac hypertrophy without affecting cardiac dysfunction in the myocyte-specific *Dsp* haplo-insufficient (*Myh6-Cre: Dsp*^W/F^) mice [109], probably because the exercise protocol used in this study is to gradually increase the workload. The limitation of this study is that it did not involve the effect of treadmill exercise on arrhythmia in elderly mice. These also demonstrate that when studying the effects of physical activity on disease progression and arrhythmia in the ACM model, the type of training plays a critical role. Hammer et al. showed that low-intensity exercise in the *PKP2*^+/-^ mouse model did not lead to fibrofatty replacements or rearrangement of gap junctions, SCD and an increase in obvious arrhythmias, calcium handling and contractility alterations of isolated myocytes caused by exercise were mostly abolished in these animals, suggesting that the low-intensity exercise seems to be beneficial even in the early, short-term stages [77]. Endurance exercise training caused *PKP-2* R735X mutant mice to have a clear RV dysfunction resembling the ACM phenotype, such as impaired global RV systolic function and RV regional wall motion abnormalities and connexin 43 delocalization at intercardiomyocyte gap junctions, suggesting that endurance exercise is a key risk factor for the development of ACM, heart failure, arrhythmias and sudden death [20, 71, 136]. Moreover, Moncayo-Arlandi et al. found that endurance training triggered the ACM phenotype in truncated PKP2 mice [72], whereas endurance exercise accelerated ACM pathogenesis in Tg-*DSP* (R2834H) mice and this event is associated with perturbed AKT1 and GSK3-β signaling [95].

##### Inflammation

It has been reported that in addition to exercise, pressure overload and inflammation are associated with ACM [75, 137–140]. The effects of exercise, pressure overload, and inflammation on the progression of *PKP2*-related diseases were studied in heterozygous *PKP2* knockout mice (*PKP2*-Hz), showing that *PKP2* haploinsufficient mice displayed reduced Ca2 + -handling-related proteins expression, such as CaV1.2, SERCA2a, AnkB, and Casq2. Pressure overload increased levels of fibrosis and impaired electrical conduction rather than structural remodeling, leading to exercise-induced pro-arrhythmic cardiac remodeling [78]. However, whether cardiac electrical remodeling and Ca^2+^-handling disturbances were directly linked in these models remains uncertain. Patients with ACM have elevated circulating levels of pro-inflammatory cytokines, such as interleukin (IL)-1β, IL-6, and tumor necrosis factor-α (TNF-α). Thus, inhibiting the complement factor C5a receptor (CD88), blocking GSK3β, activating NF-κB, and employing Bay11-7082 may be the therapeutic options of blunting inflammatory signaling [103, 134, 139, 141, 142]. Moreover, recent advances reveal that extracellular vesicle (EVs) which are secreted by cardiosphere-derived cells (CDCs) also improve cardiac function, reduce cardiac inflammation, and suppress arrhythmogenesis in ACM [91].

##### miRNAs

In the past few decades, a large number of microRNAs have been proved to play a crucial role in various disease phenotypes, including cardiovascular diseases. Recently, miRNAs have also been shown to be altered in ACM, such as miR-21-5p, and miR-135b, miR-184, miR-130a, miR-217-5p and miR-708-5p, along with miR-499-5p [90, 124, 125, 143, 144]. Yet, further studies will be necessary to reveal the potential pathophysiological roles of miRNA and underlying mechanisms in the development of ACM. Sorbin and SH3 domain-containing 2b (*SORBS2*) is another potential candidate gene for ACM susceptibility because *SORBS2* knockout mouse manifests several key features of ACM, such as right ventricular dilation, right ventricular dysfunction, spontaneous ventricular tachycardia, and premature death [108].

In addition to the cardiac inflammation, patients of ACM show a gradual fibro-adipose replacement of the ventricular myocardium. Yet, there is no pharmacological method available in clinical practice to counteract the replacement of cardiac lipogenesis. The *PLN*, tumor protein 53 apoptosis effector (PERP), and carnitine palmitoyltransferase 1β (CPT1B) involved in ion channels, apoptosis and adipogenesis play a role in the pathogenesis of ACM [145]. Some studies also demonstrated that inhibition of Wnt/β-catenin signaling can trigger adipogenesis, fibrogenesis, and apoptosis, which are characteristic of human ACM [92, 146–148]. It has been reported that the proliferator-activated receptor gamma (PPARγ), activated when Wnt/β-catenin- and Hippo-pathway are impaired, is a key regulator of ACM adipogenesis [92, 149]. Therefore, the PPARγ modulator rosiglitazone or 13-hydroxyoctadecadienoic acid (13HODE) could convert glycolysis into fatty acid metabolism to mimic ACM lipogenesis [150]. Furthermore, ACM patients show high plasma concentration of oxLDL which are major cofactors of adipogenesis. Cardiac adipogenesis and right ventricle systolic impairment are counteracted by atorvastatin treatment in a Pkp2 heterozygous knock-out mice (*Pkp2* + */* − mice) with a high-fat diet (HFD) [151]. In that study, the authors also demonstrated for the first time, that oxidative stress and oxidized lipid metabolism modulate ACM adipogenic phenotype at the cellular, mouse, and patient levels [151]. Fibrofatty infiltration only appeared in mouse models with *DSP* mutations, but not in mouse models involving *DSG2*, *DSC2*, and *JUP* [92, 93, 152, 153].

#### Mutations in Zebrafish Models

Due to the convenience of embryonic morpholine gene knockout and the adaptability of high-throughput inheritance and compound screening, zebrafish ACM models for studying *DSC2* and *JUP* mutations have also been developed [111, 154]. Furthermore, Moriartyet al. knocked down *PKP2* in zebrafish through morpholine microinjection, resulting in cardiac oedema, blood pooling, failure of the heart to loop, decreased heart rate and abnormal desmosomes in the heart, suggesting that *PKP2* is essential in cardiac development [112]. A more integrative model (zebrafish) is used to investigate the pathophysiological mechanism of ACM, which allowed us to assess the specific effect of genetic variation at the organs and organisms level. Asimaki et al. generated a zebrafish model of ACM carrying a cardiomyocyte-specific expression of the human 2057del2 mutation in the gene encoding plakoglobin to elucidate the underlying mechanisms and discover potential chemical modifiers, and SB216763 showed a remarkable ability to prevent ACM in this model [112]. Giuliodori et al. generated and validated a zebrafish model for *DSP*-associated AC using a gene knock-down approach, finding that knock-down of zebrafish *DSP* affects desmosome structure, Wnt/β-catenin, TGF-β/Smad3, and Hippo/YAP-TAZ signaling pathways [113]. Furthermore, GSK3β inhibitor rescues the AC phenotype in the zebrafish model through Wnt/β-catenin signaling, and activation of abnormal Hippo/YAP signaling pathway leads to β-catenin cytoplasmic isolation and JUP nuclear translocation [113, 142, 149, 154]. Moreau et al. established the zebrafish model to validate the effect of the *DSC2* p.R132C substitution in‐vivo, indicating that the R132C substitution impairs *DSC2* function [114].

These animal models have played a critical role in elucidating the pathophysiological mechanism of ACM and developing targeted therapies. Although many observations are consistent with those of ACM patients, clearly, animal models cannot reproduce the conditions of ACM patients due to the differences in electrophysiological functions between animal and human hearts. Therefore, the main differences in the electrophysiological characteristics of the heart of small animals and humans largely limit the translation of the results to humans. Moreover, animal models often lack research on the molecular level of gene function.

### Heterologous Expression Systems

When studying the regulatory function of a gene, normally it is necessary to verify its regulatory effect in different models. Using cell models to study its molecular mechanism is more conducive to the prediction and verification of gene function. Compared with gene knockout animals, gene knockout at the cell level has many advantages, such as lower lethality and faster construction time. Therefore, specific mutant genes identified from ACM patients transfected into cells can be helpful to understand the underlying pathophysiological mechanism and provide unique opportunities to gain insights into different forms of ACM. To date, there are many very meaningful kinds of researches related to ACM at the cellular level (Table 3).

**Table 3:** Gene, mutation, key findings, references in cell models studying ACM

Models	Gene	Mutation	Protein	Phenotype
DP-deficient HL-1 cells [92]	DSP	-	desmoplakin	Increased expression of adipogenic and fibrogenic genes and accumulation of fat droplets
The human tongue squamous cell carcinoma cell line SCC-9 [93]	DSP	V30M or Q90R, R2834H, W233X	desmoplakin	Two N-terminal DSP mutations (V30M and Q90R) affect the localization of DSP in vitro and disrupt the binding ability of DSP N- terminus
HL-1 cells [155]	PKP2	-	plakophilin-2	PKP2 deficient led to decreased INa and NaV1.5 at the site of cell contact
PKP2 deficient HL-1 cells [73]	PKP2	-	plakophilin-2	The release of ATP in cells lacking PKP2 is significantly increased; Loss of Cx43 expression drastically blunted this effect
HL-1 cells [156]	DSP	-	desmoplakin	Cx43 and Nav1.5 expression decreased following DSP silencing and then DSP suppression presented decreased INa and slowed CV
HEK-293 cells [157]	PLN	R14Del	-	sarcoplasmic reticulum Ca(2 +)-ATPase superinhibition
NRVMs [112]	-	-	-	Increased myocyte apoptosis, decreased immunoreactive signal for Cx43 at cell–cell junctions as well as a diminished immunoreactive signal for plakoglobin at cell–cell junctions and abundant signals in cell nuclei
HEK293 [158]	JUP	S39_K40insS	plakoglobin	Both DSC2-WT and DSC2-Q554X are localized at the cell membrane and remain stable
HEK293T cells [159]	PKP2	c.419C > T variant	plakophilin-2	No effect on cell proliferation
HEK293 cells [160]	DSC2	Q554X	desmocollin-2	Both DSC2-WT and DSC2-Q554X arelocalized at the cell membrane and remain stable
HEK293 [161]	DSG2	c.710 T > A, p.Leu237Ter	desmoglein-2	This null variant would decrease the expression of DSG2 gene, and the mutant DSG2 truncated protein was markedly shifted to the cytoplasm
HEK293T [162]	-	GSK3BS9A	-	A significant INa density decrease
HEK293T [163]	DSP	c.832delG	desmoplakin	Truncation of *DSP* protein, down-regulation of *JUP* and up-regulation of β-catenin expression in nuclear are observed
NRVCMs [164]	DSP		desmoplakin	Desmoplakin knock-down also impaired the Cx43 membrane localization
NRVMs [165]	PKP2		plakophilin-2	Loss of PKP2 expression led to a decrease in total Cx43 content, and Cx43 had a significant redistribution in the intracellular space
NRCMs HL-1 cells [166]	DSC2	p.E102K, p.I345T	desmocollin-2	The two missense mutations of DSC2(p.E102K mutation and p.I345T mutation) in the N-terminal domain affect the normal localization of DSC2
NRVMs [167]	PKP2	R79x and 179 fs	plakophilin-2	Mutation R79x and 179 fs of PKP2 did not alter the localization of endogenous PKP2, DP or Cx43, and R79x expression significantly reduces HSP90 levels
NRCMs [168]	-	-	-	Hypoxia/serum depletion stimulation induced significantly elevation of intracellular and extracellular HSP70
Epicardial Explant [169]	PKP2	-	plakophilin-2	Increased abundance of α-smooth muscle actin-positive cells, increased cell migration speed, and increased abundance of cell proliferation markers, increased abundance of lipid markers
NRVMs [154]	*JUP*	2057del2	plakoglobin	Pathobiological features seen in patients with ACM can be observed;
NRVMs [141]	*JUP*	2157del2	plakoglobin	Abnormal redistribution of intercalated disk proteins, release of inflammatory cytokines, myocyte apoptosis

#### HEK293 Cells

HEK293 cells are widely used in many fields and have become a powerful platform. Importantly, HEK293 cells exhibit high transfection efficiency, fast growth, efficient and flexible metabolism, and have all human post-translational modifications to produce the most similar proteins [170–173]. Asimaki and colleagues identified a mutation (S39_K40insS) of *JUP* in an ACM-affected German family, which is a novel autosomal dominant plakoglobin mutation. They reported that HEK293 cells expressing the mutant plakoglobin had a higher proliferation rate and a lower apoptosis rate [158]. However, Christensen et al. did not find that the *PKP2* c.419C > T variant has an effect on cell proliferation, indicating that *PKP2* c.419C > T lacks a functional effect [159]. Gerull et al. transfected HEK293 cells with constructs expressing human *DSC2* WT or mutant *DSC2*-Q554X, finding that both *DSC2*-WT and *DSC2*-Q554X are localized at the cell membrane and remain stable [160]. Desmoplakin knock-down (*DP*-KD) also impaired the Cx43 membrane localization in neonatal rat ventricular myocytes (NRVCMs), suggesting that *DP* regulates membrane localization [164]. To document that the current amplitude change is independent of cell-type, Riele et al. transiently transfected HEK293 cells with cDNA coding for *SCN5A*, demonstrate in that similar to the hiPSC-CMs, the current density generated by a construct containing the *SCN5A* p.Arg1898His mutation was significantly reduced compared with wild-type *SCN5A* [174]*.* Chen et al. identified that the *DSG2* gene expression is significantly decreased in a novel nonsense variant in *DSG2* (c.710 T > A, p.Leu237Ter) group, suggesting this null variant would decrease the expression of *DSG2* gene, and the mutant *DSG2* truncated protein was markedly shifted to the cytoplasm, which suggests that the nonsense variant (*DSG2 c.710 T* > *A, p.Leu237Ter*) could affect the expression and function of DSG2 protein [161]. More importantly, these results could be repeated in AC16 cell model [161]. Furthermore, Khudiakov et al. observed that GSK3B^S9A^ mutation expression did not result in a decrease of Wnt/β-catenin signaling activity but led to a significant INa density decrease in GSK3BS9A transfected HEK293T cells [162]. HEK293T cells transfected with mutant plasmids led to the truncated *DSP* mRNA and protein, upregulation of nuclear *JUP* and downregulation of β-catenin, when compared with WT. Truncation of *DSP* protein, down-regulation of *JUP* and up-regulation of β-catenin expression in nuclear but not cytoplasm are observed in the HEK293T cells transfected with *DSP c.832delG* [163].

#### HL-1

HL-1(Heart Atrial cells derived from mice) has been extensively characterized and is a valuable model system to address questions of cardiac biology at the cellular & molecular levels with a phenotype similar to adult cardiomyocytes. Garcia-Gras et al. established *DP*-deficient HL-1 cells and showed that suppression of *DP* expression led to nuclear localization of the desmosomal protein plakoglobin and a twofold reduction in canonical Wnt/beta-catenin signaling through Tcf/Lef1 transcription factors [92]. Yang et al. also demonstrated that the N-terminal mutants (V30M and Q90R) of *DSP* failed to localize to the cell membrane in the desomosome-forming cell line (the human tongue squamous cell carcinoma cell line SCC-9) and failed to bind to *JUP* [93]. HL-1 atrial myocytes maintain the electrophysiological functioning of healthy cardiomyocytes and express near natural levels of connexin43, making them ideal for this particular research [175]. *PKP-2* siRNA in HL-1 cardiomyocyte cells decreases connexin43 expression and alters its localization [176]. *PKP2* deficient led to decreased INa and NaV1.5 at the site of cell contact in HL-1-derived cells that endogenously express NaV1.5 but have *PKP2* deficiency. *PKP2* variants that reduce I_Na_ could be related to a Brugada syndrome (BrS) phenotype, even without overt structural features characteristic of ACM. Cx43 and Nav1.5 expression decreased and exhibited an abnormal distribution following *DSP* silencing in HL-1 cells and the *DSP* suppression also decreased I_Na_ and slowed CV, indicating that impaired mechanical coupling affects electrical synchrony in ACM to a great extent [156].

#### Primary Cells

What’s more, compared with cell lines, primary cells retain more biological characteristics of the original tissue, such as growth and senescence, so better cellular diseases models can be established through primary cells. Oxford et al. decreased *PKP2* expression in NRVCMs using RNA silencing technology, showing that loss of *PKP2* expression led to a decrease in total Cx43 content, and Cx43 had a significant redistribution in the intracellular space, which demonstrated that there is a molecular crosstalk between desmosomal and gap junction proteins [165]. The two missense mutations of *DSC2* (p.E102K mutation and p.I345T mutation) in the N-terminal domain affect the normal localization of DSC2 on neonatal rat cardiomyocytes and HL-1 cells [166]. Mutations R79x and 179 fs of *PKP2* did not alter the localization of endogenous PKP2, DP or Cx43, and the mutation R79x expression significantly reduces HSP90 levels, which leads to facilitated activation of myocyte apoptotic pathways [167]. Moreover, Wei et al. observed hypoxia/serum depletion stimulation induced significantly elevation of intracellular and extracellular HSP70 in NRCMs, indicating that elevated HSP70 is a feature of heart failure caused by ACM [168]. Epicardial explants after *PKP2* knockdown obtained from neonatal rat hearts increased abundance of α-smooth muscle actin-positive cells, cell migration speed, abundance of cell proliferation markers, and abundance of lipid markers, suggesting that a group of non-excitable cardiac resident cells express desmosome molecules and rely on *PKP2* expression in vitro [169]. Pathobiological features seen in patients with ACM can be observed in NRVMs expressed 2057del2 plakoglobin, which was reversed or prevented by SB216763 which is a suppressor of the disease phenotype [154]. NRVCMs with 2157del2 in *JUP* exhibits several features, such as abnormal redistribution of intercalated disk proteins, the release of inflammatory cytokines, myocyte apoptosis, which can be prevented by Bay 11–7082 (a small-molecule inhibitor of NF-κB signaling) [141]. Asimaki et al. transfected adenovirus into normal NRVCMs to express 2057del2 mutation plakoglobin, showing increased myocyte apoptosis, decreased immunoreactive signal for Cx43 at cell–cell junctions as well as diminished immunoreactive signal for plakoglobin at cell–cell junctions and abundant signals in cell nuclei, which recapitulates cardiac characteristics of ACM patients [112].

From the above review, heterologous expression systems expressing disease-specific mutations, such as HEK293 cells, HL-1 cells etc., and transgenic animal models have greatly facilitated our understanding of the pathogenic mechanisms associated with ACM. In fact, it is true that all published animal or cell models of ACM as well as the elucidation of the involved mechanisms and the applicability of experimental data have inherent limitations in studying human diseases, which have hampered the exploration of potential therapies for the management of human ACM. Similarly, human non-cardiac cell lines (such as HEK-293 or CHO cells) are not ideal to model the heart as they are different from cardiomyocytes in many aspects such as sarcomere tissue, metabolism and electrophysiology, etc. [177]. In this regard, cardiomyocytes derived from induced pluripotent stem cells have advantages over animal or human non-cardiac cells.

### Human Cardiomyocytes Derived from Induced Pluripotent Stem Cells (hiPSC-CMs)

The use of hiPSC-CMs is extremely versatile in studying basic and profound mechanisms of cardiomyopathies such as familial dilated cardiomyopathy (DCM), familial hypertrophic cardiomyopathy (HCM), catecholaminergic polymorphic ventricular tachycardia (CPVT), long QT syndrome (LQTS), short QT syndrome (SQTS), ACM and BrS [154, 178–188]. Compared with animal models and heterologous expression systems, one of the most important advantages of hiPSC-CMs is that it closely matches the genes of patients with specific diseases. The second advantage is the possibility to generate patient-specific cardiomyocytes for patient-specific investigation including mechanistic and therapeutic studies [183, 189]. Therefore, hiPSC-CMs are a significant preclinical model system for studying the genetic basis of human cardiovascular diseases (Table 4). hiPSC-CMs have been used to better understand the occurrence of arrhythmias [192, 196]. Several research groups have successfully generated iPSC-CMs from patients with hereditary cardiac ion channel diseases, such as the LQTS and BrS [182, 197–199]. hiPSC-CMs retain the patient’s genetic information and exclude the influence of environmental factors. Additionally, ACM patient-specific hiPSC-CMs that can model disease-specific abnormalities have been proved to recapitulate key characteristics observed in human disease [200].

**Table 4:** ACM genes related to experimental researches in hiPSC-CMs

Cell types	Gene	Mutation	Protein	Molecular mechanism
hiPSC-CMs [190]	DSG2	c.2358delA	desmoglein-2	(1) Decreased DSG2 expression and disrupted protein localization (2) shortened the APD and the time to reach peak calcium in DSG2^Mut^ CM was shortened, (3) altered Ca^2+^ handling and expression of immune cytokines
hiPSC-CMs [174]	*SCN5A*	R1898H	-	Reduced peak sodium current and reduced abundance of Na_V_1.5 and N-Cadherin clusters at the intercalated disc
hiPSC-CMs [191]	PKP2	-	plakophilin-2	A significant decrease in the expression of PKP2, and reduced densities of PKP2, the associated desmosomal protein plakoglobin as well as the gap-junction protein CX-43
hiPSC-CMs [192]	PKP2, JUP	-	plakophilin-2 plakoglobin	Reduced gene expression of PKP2 and plakoglobin, reduced immunofluorescence signals for these desmosomal proteins, and increased potential for adipocytic change
hiPSC-CMs [193, 194]	DSG2, DSP	H1684R	Desmoglein-2	Abnormal action potential, multiple ion channel currents dysfunctions,
hiPSC-CMs [155]	PKP2	-	plakophilin-2	A PKP2 deficit in hiPSC-CMs showed drastically reduced INa
hiPSC-CMs with TMEM43-S358L mutation [103]	TMEM43	-	-	Contractile dysfunction
iPSC-CMs carrying the *OBSCN* mutation [195]	-	OBSCN	-	(1) Intracellular calcium current increased (2) The mutant OBSCN protein and its anchor Ank1.5 protein appeared structural disorder and decreased expression (3) The gene expression of other desmosomal proteins also reduced whereas the adipogenesis pathway-related proteins such as PPARγ, C/EBPα, and FABP4 increased
hiPSC-CM derived from the DSC2 patient [114]	DSC2	-	desmocollin-2	A shortened action potential duration
hiPSC-CMs [174]	*SCN5A*	p.Arg1898His	-	The current density generated by a construct containing the *SCN5A* p.Arg1898His mutation was significantly reduced

#### Desmosomal Variants in hiPSC-CMs

In 2012, Ma et al. for the first time generated iPSC-derived cardiomyocytes from a patient with a clinical diagnosis of ACM and demonstrated significant phenotypes such as reduced gene expression of *PKP2* and *JUP*, reduced immunofluorescence signals for these desmosomal proteins, and increased potential for adipocytic change [192]. hiPSC-CMs from 2 ACM patients with *PKP2* mutations were established, displaying similar results, such as a significant decrease in the expression of *PKP2* and reduced densities of *PKP2*, the associated desmosomal protein plakoglobin as well as the gap-junction protein Cx-43, which was related to upregulation of the proadipogenic transcription factor PPAR-γ, whereas elevated estradiol levels decreased apoptosis and lipid accumulation of CMs in an in-vitro ACM model [22, 150, 191].

#### Sex Hormones Study in hiPSC-CMs

It is known that sex hormones regulate metabolic homeostasis of various cell types and the occurrence of arrhythmias [154, 201], especially testosterone which regulates adipogenesis of fat cells and is also associated with a high incidence of cardiovascular disease [202, 203]. Akdis et al. reported that in male ACM patients, increased serum testosterone levels were independently linked to major arrhythmic cardiovascular events (MACE), while in female MACE patients, estradiol levels were reduced, suggesting that testosterone worsened and estradiol improved cardiomyocyte apoptosis and lipogenesis in an induced pluripotent stem cell-derived ACM model [22]. Currently, researchers have successfully established the iPS cell line HUBUi001-A from a patient carrying the *DSP* heterozygous variants (c.104G > T p.G35V; c.5617C > T p.R1873C) and an iPSC cell line with a pathogenic heterozygous variant in *PKP2* (c.1799delA) from a patient affected by ACM [204, 205].

#### Ion Channel Dysfunction in hiPSC-CMs

Some researches related to arrhythmias in ACM have been conducted, mainly focusing on the electrophysiological properties of hiPSC-CMs. Riele et al. revealed reduced peak sodium current and reduced abundance of Na_V_1.5 and N-Cadherin clusters at the intercalated disc, indicating that Nav1.5 and adhesion molecules are in a functional complex, and Na_v_1.5 dysfunction may contribute to ACM [174]. El-Battrawy et al. reported that ACM-hiPSC-CMs carrying a *DSG2* gene missense variant (G to A substitution at nucleotide p.Gly638Arg) showed an abnormal action potential with reduced APA and *V*_max_, multiple ion channel current dysfunctions such as reduced I_Na_, I_to_, I_SK_, I_KATP_, I_NCX_, and enhanced I_Kr_, and also showed that ion channels were more sensitive to adrenergic stimulation, suggesting that multiple ion channel dysfunction and increased sensitivity to adrenergic stimulation are associated with arrhythmias in ACM patients [193]. Buljubasic et al. demonstrated that both NDPK-B and SK4 expressions were elevated in ACM-hiPSC-CMs with the same mutation and that recombinant NDPK-B enhanced I_SK4_, cell automaticity and arrhythmic events, whereas protein histidine phosphatase 1 (PHP-1), a counter actor of NDPK-B, prevented the NDPK-B effect, suggesting possible involvement of NDPKB/SK4 in arrhythmogenesis of ACM with *DSG2* mutations [206]. Moreover, hiPSC-CM derived from the *DSC2* patient showed that reduced Ca^2+^ current density and increased K^+^ current density led to a shortened action potential duration (APD), which may be used to elucidate the abnormal repolarization dynamics in ACM patients [114].

hiPSC-CMs with the p.S358L variant in *TMEM43* also showed contractile dysfunction, which partially recovered after GSK3β inhibition [103]. There are no reports indicating that variants in the *OBSCN* gene cause ACM. Thus, Chen et al. generated iPSC-CMs isolated from an ACM patient carrying a variant in the *OBSCN* gene, showing that the calcium current increased, the structure of mutant OBSCN protein and its anchor Ank1.5 protein structure was disordered and the expression of both proteins together with other desmosomal proteins was reduced, whereas the adipogenesis pathway-related proteins such as PPARγ, C/EBPα, and FABP4 were increased, which may explain the fibrofatty replacement of the myocardium and calcium channel-related myocardial contraction abnormalities in ACM patients [195]. Additionally, Hawthorne et al. established a novel hiPSC-CM model derived from ACM patients with a c.2358delA variant in *DSG2*, confirming that *DSG2*^Mut^ CMs harbored decreased *DSG2* expression and disrupted protein localization, whereas the expression or localization of other key desmosome components did not change significantly [190]. At the same time, the APD and the time to reach peak calcium in *DSG2*^Mut^ CMs were shortened, and the Ca^2+^ handling and expression of immune cytokines were altered [190]. Similarly, hiPSC-CMs from a patient with a *PKP2* deficit showed drastically reduced I_Na_, whereas transfection of wild-type *PKP2* can restore these deficits [155]. Khudiakov et al. generated the hiPSC-CMs model of ACM with *PKP2* genetic variants c.354delT and p.Lys859Arg to study its molecular and functional role, displaying that after inhibiting GSK3β, sodium current was restored through Wnt/β-catenin-independent mechanisms [162, 207].

hiPSCs derived from patients with specific variants can be frozen, stored, and used as an in vitro model of ACM, providing structure and function-based data for the development of new therapeutic applications. The pathogenic role of a variant can be confirmed by gene editing. It was demonstrated that repairing the gene variant could revert the disease-phenotype in the *DSC2* hiPSC‐CMs to normal state, and in addition, sotalol improved electrical activity as well as mechanical function, and flecainide normalized the frequency of spontaneous Ca^2+^ transients and the occurrence of Ca^2+^ sparks, which provide a rationale for their therapeutic application in ACM [114]. The same gene, even sometimes the same genetic variation can also cause totally different clinical characteristics, indicating that the disease entity results from multiple disease-causing factors. In addition to the genetic causes, a host of other factors, such as age, environment, genetic background, complications together with epigenetic factors will also affect the occurrence, progression, and prognosis of ACM [208].

#### Limitations of hiPSC-CMs Models

hiPSC-CMs exhibit a relatively immature phenotype and are more depolarized than adult ventricular cardiomyocytes in a resting state, which will affect the dynamics of voltage-gated ion channels and change the excitability [209]. In typical monolayer culture, they will not exhibit an elongated morphology or form fully organized insertion discs, which may affect the level and spatial distribution of desmosomal protein expression. Now more chemical, genetic, and biomechanical approaches are developed to promote the maturation of cardiomyocytes, such as the incorporation of CMs into 3D tissue constructs, bioelectrical stimulation, mechanical stretch, biochemical stimulation, and long-term culture [210–214]. For example, Liu et al. first reported that PGC-1α activator ZLN005 promotes the maturation of mitochondrial biology and energy metabolism, enhances structural maturity including sarcomere length, increases CX43 expression, enhances electrical activity, improves Ca^2+^ handling and electrophysiological characteristics to promote the maturation of hPSC-CMs [215]. Furthermore, Miki et al. identified that ERRγ agonists make hiPSC-CM be larger in size, possess longer sarcomere length, present transverse tubules, and enhance metabolic function, contraction and electrical properties, which is consistent with the characteristics of the mature neonatal CMs and contributes to disease modeling and regenerative medicine [216]. The combinations of hiPSC-derived CMs, cardiac fibroblasts (CFs), and cardiac endothelial cells can also promote the maturation of scaffold-free three-dimensional microtissues (MTs) [217]. A better hiPSC-CM platform that is more similar to native cardiomyocytes will be available in the near future.

## Conclusions and Future Perspectives

ACM is a genetic disease of the myocardium, which is characterized by ventricular arrhythmias and SCD, right ventricular dysfunction, and subsequent progressive heart failure. To date, some animals such as mice, dogs, and cats and cell models have been used to study clinical features of ACM. Each model has its unique advantages and limitations (Table 5). Transgenic animals are increasingly used in in-vivo experiments to examine the effects of gene functions, their regulation, or genetic changes on disease development. The health of many genetically modified animals has been affected by genetic modification, regardless of the procedures performed on them. Therefore, some researchers have proposed non-animal transgenic methods, such as cell transfection technology. However, cell transfection technology has several disadvantages such as low cell transfection efficiency and high cell death rate, especially the primary cultured cells. Some diseases lack appropriate animal models or in vitro models. Therefore, hiPSC-CMs become a valuable model for studying the pathological mechanism in ACM. Although hiPSC-CMs are relatively less mature than isolated adult ventricular cardiomyocytes, such as disorganized sarcomeric filament, lack of t-tubular network, polygonal shapes, and rhythmic automaticity, more useful approaches have been studied to promote the maturity of hiPSC-CMs. There is no doubt that the advantages of this cell type outweigh the disadvantages, e. g. generating patient-specific cells, editing the genome of healthy and disease cells to insert or correct mutations/variants.

**Table 5:** Advantages and disadvantages of ACM preclinical models

Models	Advantages	Disadvantages
Spontaneous animal models	Closer to nature human diseases; High application value	Limited variety and low prevalence
Transgenic mice	Mimic clinical features of human ACM; easier to feed than any other animal; ACM can be studied in different physiological environments and developmental stages	High cost and time consuming; energy, beat rate, and expression of key ion channels differ from humans
Heterologous expression	Studying cellular mechanisms of ACM; high transfection efficiency, fast growth, efficient and flexible metabolism, and have all human post-translational modifications	Lack of macromolecular ion channel complexes; different from cardiomyocytes in sarcomere tissue, metabolism and electrophysiology
Primary cells	Normal cell morphology and important markers and functions; retain more biological characteristics of the original tissue; Express disease-specific mutations	a finite lifespan and limited expansion capacity
hiPSC-CMs	Easier to generate patient-specific cardiomyocytes; exclude the influence of environmental factors; model disease-specific abnormalities	Exhibit a relatively immature phenotype; lack of elongated form and fully organized insertion disk

Moreover, genome editing tools including clustered regularly interspaced short palindromic repeats (CRISPR/Cas9), transcription activator-like effector nucleases (TALEN) etc. have largely facilitated the applications of hiPSC-CMs by altering gene expression and correct genetic variation [218–222]. Hence, hiPSC-CMs combined with gene-editing technology have become a powerful tool for studying the pathophysiological mechanism of ACM and a substitute for animal models. In general, hiPSC-CMs are more widely used in preclinical research and regenerative medicine. Currently, different methods have been adopted to study the pathogenesis of ACM, including in vitro cell and tissue models and in vivo models. However, it is still challenging to fully reproduce the clinicopathological features of ACM in a laboratory environment. Therefore, more work needs to be done to promote the innovation of advanced ACM models to reproduce these subtle physiological effects to uncover the pathology and clinical findings of ACM.

Currently, the source of the correlation between genetic variation and phenotype in ACM can be the direct, major, and mixed impact of variation. For the complex traits present in ACM, it is still challenging to identify all the causal variants and clarify their underlying mechanisms. Male and multiple gene variants, which are important factors, affect the prognosis of ACM [8, 21]. Current effective treatments for ACM include lifestyle changes, traditional pharmacological therapy, catheter ablation, ICD, and heart transplantation. However, every treatment has its limitations. Numerous approaches such as using cardiac stem cells to regenerate cardiomyocytes or collecting cardiac progenitor cells to produce beneficial factors are rapidly entering clinical trials to solve various forms of cardiomyopathy [223]. WES and whole-genome sequencing (WGS), multi-omics technology and hiPSC-CMs are new tools for studying the genetics of ACM, which provide a new platform for unraveling the complex molecular interactions of ACM and clinical management. Therefore, an in-depth understanding of gene mutation-phenotype association and individualized treatment are the prerequisites for achieving precision medicine in ACM.

## Data Availability

Not applicable
